# Management of submacular hemorrhage: from a case report to a comprehensive review of current treatment strategies

**DOI:** 10.1007/s10792-026-04011-z

**Published:** 2026-03-06

**Authors:** Federico Giannuzzi, Lorenzo Hu, Mattia Cusato, Valentina Cestrone, Umberto De Vico, Carmela Grazia Caputo, Clara Rizzo, Ludovica Di Fede, Matteo Mario Carlà, Emanuele Crincoli, Stanislao Rizzo

**Affiliations:** 1 Ophthalmology Unit, Fondazione Policlinico Universitario A. Gemelli, IRCCS, Largo A. Gemelli, 8, Rome, Italy; 2 Università Cattolica del Sacro Cuore, Rome, Italy; 3 Department of Ophthalmology, Hospital Foundation Adolphe De Rothschild, Paris, France; 4 Department of Surgical, Medical and Molecular Pathology and Critical Care Medicine, University of Pisa, Pisa, Italy

## Abstract

**Purpose:**

To evaluate current pharmacological and surgical strategies for managing submacular hemorrhage (SMH), a vision-threatening complication which primarily occurs with macular neovascularization in age-related macular degeneration (AMD).

**Methods:**

The research involved a literature review of recent studies about SMH treatment methods including anti-vascular endothelial growth factor (VEGF) therapy, tissue plasminogen activator (tPA), pneumatic displacement and pars plana vitrectomy techniques through meta-analyses, comparative studies and case series.

**Results:**

SMH treatment is guided by hemorrhage size: small (≥ 1 to < 4 disc diameters), medium (≥ 4 disc diameters within the temporal arcade), massive (exceeding temporal arcades). Pharmacological management includes anti-VEGF monotherapy, which demonstrates efficacy comparable to surgical interventions for smaller hemorrhages, while offering a superior safety profile. Combined of tPA and anti-VEGF therapy achieves an 86% displacement success rate, with comparable efficacy between subretinal and intravitreal delivery methods. Surgical methods include pneumatic displacement, which achieves 85–100% efficacy in displacement and 45–80% rates of visual improvement, whereas pars plana vitrectomy is preferred for cases involving dense, organized hemorrhages. Retrospective studies indicate that outcomes are primarily influenced by patient-specific factors, such as hemorrhage size and baseline visual acuity, rather than the treatment modality employed. Intervention within 7 to 14 days has been shown to enhance outcomes, particularly when using a stepwise protocol that begins with less invasive techniques and escalates only as necessary.

**Conclusions:**

Modern SMH management emphasizes individualized, time-sensitive treatment based on hemorrhage characteristics. A stepwise approach, beginning with pharmacological therapy and moving to surgery only when necessary, tends to offer the best balance between visual recovery and safety. Timely diagnosis and intervention are essential factors for success due to the rapid damage of photoreceptors occurring within 24–72 h of onset.

## Introduction

Submacular hemorrhage (SMH) represents a rare yet vision-threatening condition characterized by the accumulation of blood between the neurosensory retina and retinal pigment epithelium (RPE) [1]. It commonly arises in the setting of choroidal neovascularization (CNV), including age-related macular degeneration (AMD), polypoidal choroidal vasculopathy (PCV), high myopia, and angioid streaks [2]. Other causes include trauma, particularly choroidal rupture, retinal artery macroaneurysms (RAM), iatrogenic causes during surgical procedures, and inflammatory and systemic conditions such as coagulopathies or sickle cell disease [3]. The differential diagnosis for SMH encompasses a broad spectrum of pathologies, with choroidal neovascularization being the most frequent underlying cause. In AMD patients, CNV can manifest as three distinct forms: Type 1 (sub-RPE), Type 2 (subretinal), or Type 3 (retinal angiomatous proliferation), each carrying distinct hemorrhagic risks [4]. Despite various proposed classification systems, no consensus on SMH categorization has been reached. What is clear, however, is that its pathophysiological impact is both severe and time-sensitive. The central vision loss it causes is often profound and permanent. Irreversible damage to photoreceptors manifests within 1–3 days via various mechanisms, resulting in photoreceptor apoptosis that disrupts the bidirectional nutrient exchange between the RPE and the neurosensory retina. This process is subsequently accompanied by clot formation and toxic damage from iron, hemosiderin, and ferritin [5]. Clinical studies document progressive visual deterioration in 80% of AMD-related SMH cases over two years, with mean visual acuity declining from 20/240 to 20/1250 [6]. Management depends on the hemorrhage’s size and extent. For small submacular hemorrhages, less invasive options including intravitreal recombinant tissue plasminogen activator (rtPA), anti-vascular endothelial growth factor (VEGF) agents and gas, such as sulfur hexafluoride (SF6), may be used individually or in combination, depending on the clinical scenario [7]. In medium or large SMH, surgical intervention is often required. These may include pars plana vitrectomy (PPV) with direct clot evacuation, subretinal tPA injection, pneumatic displacement with expansile gases, and RPE-choroid patch grafting [8]. Recent studies have shown that PPV combined with subretinal tPA and gas can improve visual outcomes, and innovative techniques such as limited retinotomy may further stabilize or enhance vision [9]. This case report reviews the current landscape of SMH management and its challenges. Through a complex clinical scenario, we aim to illustrate how emerging technologies can refine surgical approaches and potentially improve outcomes in difficult submacular hemorrhage cases.

## Classification and pathophysiology

SMH classification depends on hemorrhage size and has been refined to provide better prognostic information [10]:

- **Small-sized** ≥ 1 to < 4 disc diameters (DD), often fovea-centered
- **Medium-sized** ≥ 4 DD, not extending beyond temporal vascular arcade
- **Massive-sized** extends beyond temporal arcades, often involving > 50% of macular area

Although disc diameter based classification provides useful initial framework, recent studies have proposed additional classification parameters including hemorrhage thickness, duration since onset, and presence of associated subretinal fluid or pigment epithelial detachment. These factors significantly influence treatment selection and prognosis, with thick hemorrhages (> 500 μm on optical coherence tomography—OCT) showing poorer responses to pharmacological interventions alone [6, 10, 11].

## Pathophysiological mechanisms

Submacular hemorrhage (SMH) initiates a sequence of events that progressively damage the retina. The initial hemorrhage creates a physical layer between photoreceptors and RPE, disrupting the bidirectional nutrient exchange essential for photoreceptor survival [12, 13]. Within hours, the hemorrhage undergoes clot formation, resulting in a more organized and compact barrier that further impairs retinal function.

Mechanical blockade, however, is only one factor. As erythrocytes break down, free iron accumulates in the subretinal space, catalyzing reactive oxygen species, promoting lipid peroxidation, and leaving hemosiderin and ferritin deposits that may linger after the hemorrhage has resolved. The ensuing oxidative stress disrupts mitochondrial activity, damages DNA, and triggers photoreceptor apoptosis, accounting for the strong association between visual outcome and the duration of hemorrhage [14, 15].

The inflammatory response to SMH contributes to tissue damage. Complement activation, cytokine release, and inflammatory cell infiltration produce a negative microenvironment that accelerate photoreceptor degeneration [16]. This inflammatory cascade also contributes to the formation of fibrotic tissue, which can create permanent architectural distortion even after successful hemorrhage clearance [12].

## Multimodal imaging approach 

Modern evaluation of SMH requires comprehensive multimodal imaging to fully characterize hemorrhage extent and guide treatment decisions. OCT provides detailed cross-sectional anatomy, revealing hemorrhage thickness, retinal layer integrity, and associated fluid [17]. Spectral-domain OCT can further distinguish between subretinal and sub-RPE hemorrhage components, information crucial for treatment planning [18].

Fluorescein angiography remains essential for identifying underlying choroidal neovascularization patterns, though interpretation can be challenging in the presence of dense hemorrhage. Indocyanine green angiography proves particularly valuable in cases of suspected polypoidal choroidal vasculopathy, where hemorrhage may obscure characteristic polypoidal lesions on conventional angiography [19, 20]. In a series of 51 eyes studied with ICGA, the causes of SMH were wet AMD (≈ 53%), PCV (≈ 37%), RAM (≈ 6%), and lacquer cracks [21].

OCT angiography (OCTA) has emerged as a valuable non-invasive tool for detecting residual neovascular flow in areas not obscured by hemorrhage.[22] This technology helps identify active neovascularization requiring ongoing anti-VEGF therapy and can guide treatment decisions in cases where conventional angiography is inconclusive [23].

**Fig. 1:**
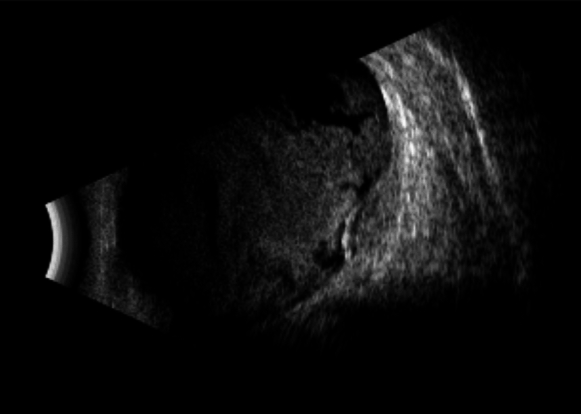
B-scan ultrasonography (US), showing a 2–3 mm thickened, heterogeneously layered submacular lesion with associated vitreous opacities, no signs of vitreoretinal traction. Findings suggest evolving age-related macular degeneration (AMD) and secondary vitreous opacities

On B-scan imaging, submacular hemorrhage appears as raised retinal tissue with echogenic patterns between the neurosensory retina and the retinal pigment epithelium (Fig. 1). Vertical macular scans show hemorrhage with the posterior lens surface on the left and the macula on the right. The diagnostic concordance with surgical outcomes is 78% with standardized methods and expert ocular ultrasonographers [24].

In cases of acute submacular bleeding, vitrectomy is necessary. It accurately detects trauma-associated retinal disease. Ultrasound may reveal hemorrhagic opacity that blocks OCT and fluorescein angiography. Ultrasound improves subretinal tPA and pneumatic positioning displacement surgery planning [25]. Serial B-scans are performed to evaluate the resolution of hemorrhages and the outcomes of treatment.

## Prognostic factors and risk stratification

Patient-specific factors significantly influence SMH prognosis and should guide treatment selection. Age represents a crucial variable, with younger patients generally showing better visual recovery potential.^26^ Baseline visual acuity serves as a strong predictor of prognosis, with eyes presenting with 20/70 or better usually having better outcomes [26].

The presence of subfoveal fibrosis or RPE atrophy dramatically worsens prognosis, as these changes indicate irreversible damage to the neurosensory retina-RPE interface. Conversely, maintained ellipsoid zone integrity on OCT correlates with better visual recovery potential, even in cases of large hemorrhages [27, 28]. Studies demonstrate that the preoperative presence of detectable ellipsoid layers and lower height of SMH may predict good visual prognosis [29].

Systemic factors including anticoagulation status, bleeding disorders, and cardiovascular disease influence both SMH risk and treatment safety. Patients on anticoagulation therapy require careful risk–benefit analysis, as both continued therapy and temporary discontinuation carry significant risks [30].

## Treatment strategies

### Pharmacological management

#### Anti-VEGF: mechanisms and optimization

Anti-VEGF agents contribute to SMH management through several complementary actions. In addition to their primary anti-angiogenic effects, these medications reduce vascular permeability, potentially decreasing additional hemorrhage risk from unstable neovascular complexes. The anti-inflammatory effect could also reduce the inflammatory cascade initiated by subretinal blood.

Which molecule to choose remains debated. Bevacizumab, ranibizumab, and aflibercept have all showed efficacy in SMH management [31, 32]. Small series suggest that aflibercept's longer half-life and broader VEGF binding profile may offer advantages in cases with significant inflammatory components [33]. However, head-to-head comparisons remain limited, and agent selection often depends on institutional preferences and availability [34].

Dosing strategies have evolved beyond standard protocols, with some centers employing intensive initial dosing (monthly for 3–6 months) followed by treat-and-extend regimens. The rationale for intensive therapy lies in maximizing early hemorrhage reabsorption while addressing underlying neovascularization activity [35].

#### Tissue plasminogen activator: pharmacokinetics and optimization

tPA facilitates enzymatic clot liquefaction through plasminogen activation, turning a consolidated hemorrhage into a fluid mass that can be displaced. The medication's efficacy depends heavily on timing, with optimal results achieved within 7–14 days of onset when clot organization remains limited [36, 37].

Dosing considerations balance efficacy with safety, as higher doses (≥ 100 μg) have been associated with retinal toxicity in animal models [13, 38]. Standard intravitreal dosing ranges from 25–50 μg, while subretinal injection typically employs 12.5–25 μg because of direct tissue contact. The medication's half-life of approximately 4–6 h in vitreous suggests that timing of adjunctive procedures (gas injection, positioning) should be optimized accordingly.

Recent research into modified tPA formulations, including sustained-release preparations and combination with anti-VEGF agents in single injections, represent promising options for improving treatment convenience and efficacy.

#### Combined pharmacological approaches

Combining intravitreal tissue plasminogen activator (tPA) with an anti‑VEGF agent targets several levels of SMH pathology at once. Meta-analysis by Veritti et al. of 781 eyes demonstrated 86% successful displacement with combined therapy, representing a significant improvement over monotherapy approaches [39]. The synergistic effects are probably due to tPA-induced clot dissolution, which facilitate anti-VEGF diffusion into the underlying neovascular tissue.

Injection timing has been refined based on drug pharmacokinetic. Many surgeons deliver the injections during the same session, the reason is to overlap the window of tPA activity with the initial therapeutic effect of anti-VEGF agents. However, some centers prefer sequential administration, allowing tPA to achieve maximal clot liquefaction before anti-VEGF injection.

## Surgical management

### Pneumatic displacement: technical considerations and refinements

Pneumatic displacement uses an expanding intraocular gas bubble to mechanically shift blood from the fovea. The selection of gas is important: sulfur hexafluoride (SF₆) provides 2–3 weeks of tamponade, whereas perfluoropropane (C₃F₈) provides a longer duration of 6–8 weeks, and may be preferred in cases with dense or organized hemorrhages that require prolonged displacement [40].

Patient positioning protocols have been tailored to optimize displacement direction. Traditional prone positioning may be modified based on hemorrhage location, with face-down positioning for inferior displacement and lateral positioning for temporal displacement. Some centers employ dynamic positioning protocols, alternating between positions to maximize displacement efficacy [41, 42].

In pseudophakic eyes, an anterior chamber paracentesis prevents intraocular pressure rise during gas expansion. This is particularly important in eyes with compromised outflow or pre-existing glaucoma [43].

### Pars plana vitrectomy: advanced techniques and innovations

Modern vitrectomy techniques for SMH aims to clear the clot while minimizing surgical trauma. Small-gauge instrumentation (25–27 gauge) reduce wound size and speed recovery, and the use of wide-field viewing systems allows a comprehensive view of hemorrhage extent and associated pathology.

Subretinal injection techniques have been refined with the introduction of 36–41 gauge cannulas, allowing precise tPA delivery directly to the hemorrhage site (Table 1). The subretinal approach theoretically offers advantages in terms of higher local concentration and longer contact time, however clinical studies have not consistently showed superiority over intravitreal administration [44].

**Table 1:** SMH treatment approaches based on hemorrhage size

SMH characteristic	Definition	Preferred techniques
Small-sized	≥ 1 to < 4 DD	• Pharmacologic treatment (Anti-VEGF, tPA) ± Pneumatic displacement in select cases • Observation (in select cases)
Medium-sized	≥ 4 DD, not extending beyond temporal arcade	Combination therapy is preferred: • Anti-VEGF + intravitreal tPA + pneumatic displacement (SF_6_ or C₃F_8_) • If ineffective: PPV + subretinal tPA
Massive-sized	Extends beyond temporal arcades Involves > 50% of macular area	Surgical treatment may be necessary: • PPV + subretinal tPA + anti-VEGF + pneumatic displacement (C_3_F_8_ preferred)

DD, disc diameter; VEGF, vascular endothelial growth factor; tPA, tissue plasminogen activator, SF_6_, sulfur exafluoride; C_3_F_8_, octafluoropropane; PPV, pars plana vitrectomy

Real-time intraoperative OCT complements these techniques, allowing real-time visualization of retinal layers and confirming hemorrhage clearance during surgery. This technology enables surgeons to optimize tPA injection location and volume while minimizing retinal trauma.

## Case report

Here, we present a case that illustrates the complexity of SMH treatment. The patient followed a stepwise management strategy beginning with pharmacologic intervention with anti-VEGF, tPA, and gas, followed by vitrectomy. The final step employed a novel surgical innovation incorporating a human amniotic membrane (hAM) patch to seal the retinotomy and promote retinal recovery.

A 67-year-old male presented in our clinic with sudden, painless visual loss in the left eye (LE). Symptoms had begun approximately 3 days before presentation. His ophthalmic history was notable for bilateral AMD with three prior intravitreal injections of bevacizumab in the LE, the most recent administered three months earlier.

On general history, the patient presented an insulin-dependent type 2 diabetes mellitus (treated with insulin and metformin) and ischemic heart disease (on bisoprolol and ramipril). His systemic medications included cardioaspirin, which had been suspended prior to surgical planning.

**Fig. 2:**
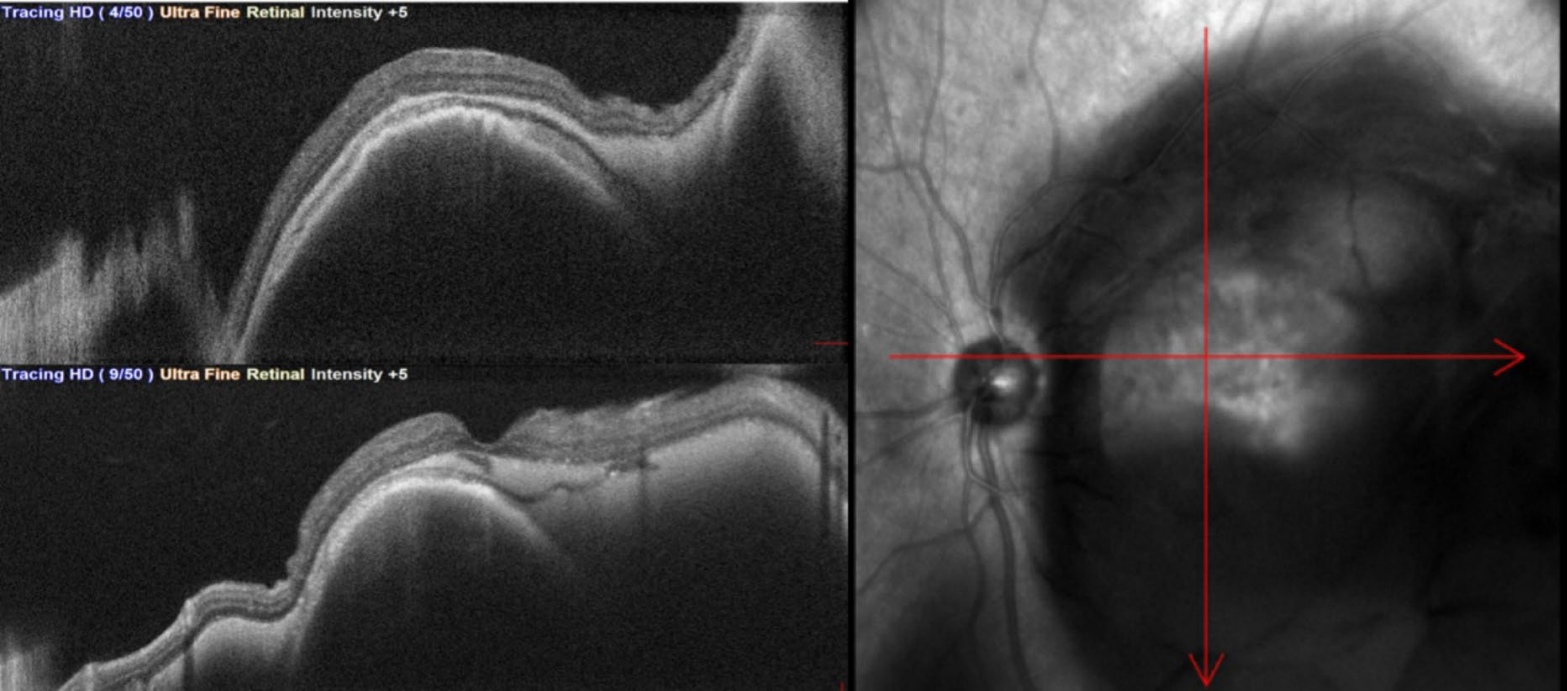
Optical Coherence tomography showing a deep subretinal lesion accompanied by retinal architectural deformation

At the time of presentation, best-corrected visual acuity (BCVA) was 0.2/10 in RE and hand motion (HM) in LE. Fundus examination of the LE revealed a large submacular hemorrhage extending beyond the vascular arcades. An OCT exam was performed, showing a dome-shaped subretinal mass (Fig. 2). A diagnosis of submacular hemorrhage (SMH) associated with neovascular AMD was established.

An initial pharmacologic approach was attempted. The patient underwent intravitreal injection of tissue plasminogen activator (tPA), bevacizumab, and pure sulfur hexafluoride (SF_6_) gas following anterior chamber paracentesis. The procedure was performed under peribulbar anesthesia. Post-procedure care included topical antibiotics and corticosteroids, ocular patching, and intravenous mannitol to reduce intraocular pressure. Post-operative follow-up visits were scheduled at day 1, week 1, and month 1, with additional visits as clinically indicated.

On postoperative visit (week 1), fundus examination of the LE was not possible due to dense vitreous hemorrhage. Ocular ultrasonography revealed significant vitreous opacities with a flat retina and a macular elevation of approximately 2 mm (Fig. 1).

**Fig. 3:**
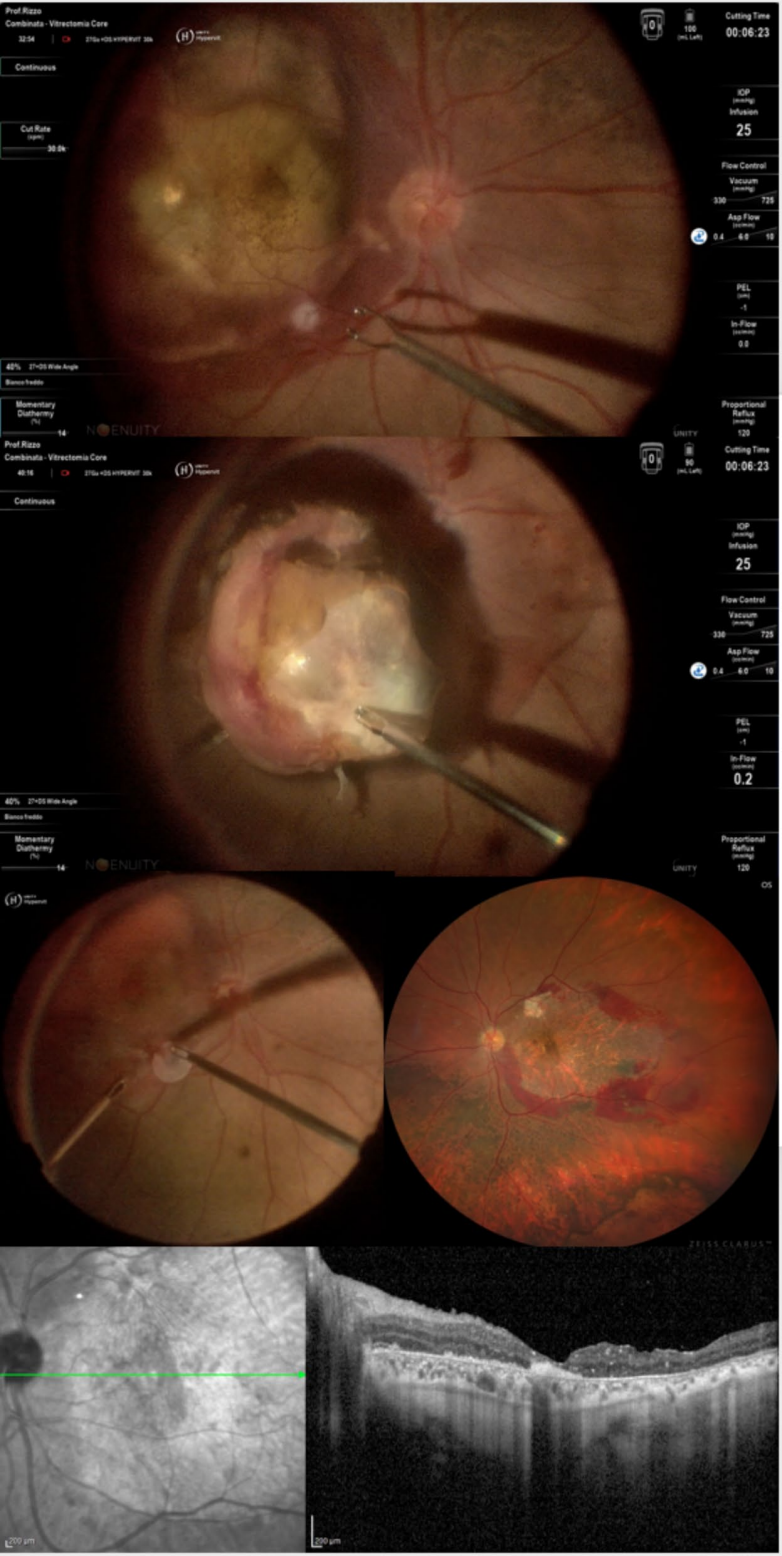
Comprehensive surgical management of submacular hemorrhage: (Top) Intraoperative images showing endodiathermy application near the superotemporal vascular arcade to create a retinotomy for subretinal hemorrhage access, followed by clot evacuation using vitreoretinal forceps; (Middle left) Placement of human amniotic membrane (hAM) patch over the retinotomy site with perfluorocarbon liquid stabilization; (Middle right) Postoperative widefield fundus photograph demonstrating resolution of submacular hemorrhage with central macular atrophy, residual pigmentary changes, and visible white hAM graft remnant near the superotemporal arcade; (Bottom) Pre- and postoperative OCT B-scans showing complete hemorrhage clearance with partial restoration of foveal contour and persistent retinal layer disruption indicating residual photoreceptor and RPE damage

Thus, the patient underwent a combined pars plana vitrectomy and subretinal clot evacuation, incorporating a novel approach using a human amniotic membrane (hAM) patch to close the retinotomy. The surgery began with phacoemulsification and intraocular lens implantation to improve visualization of the posterior segment. Subsequently, three 27-gauge transconjunctival trocars were placed via pars plana, and a chandelier light was positioned at 12 o’clock. Central and peripheral vitrectomy was performed to clear dense vitreous hemorrhage, revealing a persistent submacular hemorrhage. A small retinotomy was created using endodiathermy near the temporal macula, and the subretinal clot was gently evacuated using vitreoretinal forceps (Fig. 3). A perfluorocarbon liquid (PFCL) bubble was injected over the posterior pole to stabilize the retina during the maneuver. A hAM patch was then positioned over the retinotomy to promote closure and support tissue healing (Fig. 3). PFCL-air exchange was carried out, followed by tamponade with 20% SF_6_ gas. During the routine 1-month follow up the patient presented an improved visual acuity of 20/800. Wide field retinography was performed and showed absence of complications like retinal detachment or endophthalmitis, the resolution of the submacular hemorrhage, with persistence of subretinal hemorrhages on the vascular arcades (Fig. 3). Macular OCT showed a partial restoration of the retinal layers, with almost complete loss of the photoreceptor’s layers and the presence of hyper-reflective material in the foveal area (Fig. 3).

## Emerging and innovative techniques

### Subretinal drug delivery systems

Recent developments in sustained-release drug delivery systems offer potential advantages for SMH management. Biodegradable implants containing anti-VEGF agents or anti-inflammatory medications could provide prolonged therapeutic levels while reducing injection frequency [45].

These systems may be particularly valuable in cases requiring extended treatment courses or in patients with poor compliance.

Nanoparticle-based delivery systems represent another promising avenue, potentially allowing targeted drug delivery to specific retinal layers and enhancing drug penetration through organized hemorrhages while reducing systemic exposure [46].

### Regenerative medicine approaches

The application of regenerative medicine techniques to SMH management remains investigational but shows promise in addressing the underlying retinal damage. Human amniotic membrane patches have been employed to support retinal healing after clot evacuation, potentially reducing fibrotic complications [47].

Stem cell therapy approaches, including intravitreal injection of mesenchymal stem cells or retinal progenitor cells, aim to replace damaged photoreceptors and RPE. While clinical experience remains limited, animal studies suggest potential benefits in terms of visual recovery and reduced inflammation [48].

### Novel surgical approaches

Robotic-assisted surgery represents an emerging frontier in vitreoretinal procedures, potentially offering enhanced precision in subretinal manipulations. These systems could allow more controlled tPA injection and hemorrhage evacuation while reducing surgeon fatigue and improving consistency. Laser adjuncts, such as subthreshold diode therapy, are also under investigation; by modulating chorioretinal fluid dynamics, they may speed blood resorption and thus serve as minimally invasive complements to conventional treatments.

## Clinical outcomes and evidence synthesis

### Comparative effectiveness research

Large-scale comparative studies have provided insights into optimal SMH management strategies. Hillenmayer et al. evaluated five surgical strategies in 201 eyes demonstrating modest overall improvement (1.7–1.4 logMAR), with only stepwise protocols achieving statistical significance [49]. Therefore, a graduated step-wise treatment approach may improve outcomes while limiting complications.

A multicenter study by Murphy et al. of 88 eyes found that baseline factors, such as hemorrhage size and presenting visual acuity, were stronger determinants of prognosis than the treatment chosen [50]. This suggests that careful patient selection may be more important than specific technique choice.

### Long-term outcomes and quality of life

Beyond visual acuity measurements, modern SMH management must consider broader impacts on patient quality of life. Studies that use validated, vision-related quality of life instruments show that successful SMH treatment can significantly improve reading ability, mobility, and psychological well-being [51]. Metamorphopsia represents a common long-term sequela of SMH, occurring in 60–80% of patients regardless of treatment modality even after successful hemorrhage clearance and visual acuity improvement [52].

### Economic considerations

The economic impact of SMH management extends beyond direct treatment costs to include long-term care needs and productivity losses. Cost-effectiveness analyses suggest that early intervention with less invasive techniques may offer superior economic outcomes compared to delayed surgical intervention [53]. However, these analyses must consider the higher failure rates of conservative approaches and potential need for additional treatments.

## Future directions and research priorities

### Biomarker development

The identification of prognostic biomarkers could revolutionize SMH management by enabling more precise treatment selection. Potential biomarkers include inflammatory mediators in vitreous or aqueous humor, genetic polymorphisms affecting drug metabolism, and imaging-based markers of retinal health. In parallel, artificial‑intelligence tools trained on multimodal images are being explored for automated SMH detection, lesion mapping and, ultimately, prediction of treatment response [54].

### Therapeutic innovation

Gene therapy approaches for SMH management remain theoretical but could address underlying neovascular pathology more comprehensively than current treatments. These approaches might be particularly valuable in cases with recurrent hemorrhage or persistent neovascular activity. Drug development is also advancing: next‑generation anti‑VEGF molecules with longer intra‑ocular residence times, and bispecific antibodies that act on multiple pathways, could provide broader control in hemorrhages complicated by inflammation.

### Clinical trial priorities

Future clinical trials should focus on head-to-head comparisons of current treatment modalities with standardized outcome measures and adequate follow-up periods. Composite outcome measures that include visual acuity, anatomical resolution and patient‑reported quality‑of‑life scores would enhance both scientific rigour and clinical relevance. Trials incorporating genetic or biomarker‑guided stratification may further clarify which patients benefit most from each approach.

## Conclusion

Modern SMH management has evolved from a purely surgical discipline to a multidisciplinary field incorporating pharmacological, surgical, and emerging therapeutic approaches. The emphasis on individualized treatment based on hemorrhage characteristics and patient-specific factors represents a significant advancement in clinical care. While surgical and pharmacological approaches show comparable efficacy in many cases, stepwise protocols beginning with less invasive techniques appear to optimize outcomes while minimizing surgical risks.

Early intervention remains critical, given the rapid photoreceptor toxicity that occurs within 24–72 h of onset. This time sensitivity translates into the need for efficient diagnostic protocols and rapid intervention, often within the same or next day.

Future advances in SMH management will likely focus on personalized treatment approaches, novel drug delivery systems, and regenerative medicine techniques. The integration of artificial intelligence and advanced imaging technologies promises to improve both diagnostic accuracy and treatment precision.

The move toward less invasive, more targeted therapies promises improved outcomes with reduced complications. However, the fundamental principles of early diagnosis, prompt intervention, and individualized treatment planning remain central to successful SMH management and will continue to guide clinical practice as new technologies and techniques emerge.

## Data Availability

The data that support the findings of this study are available from the corresponding author, Lorenzo Hu, upon reasonable request.
